# 

**Published:** 1861-10

**Authors:** 


### DENTAL REGISTER

### OF THE WEST.

EDITED BY

J. TAFT AND GEO. WATT.

OCTOBER—1861.

**MONTHLY:**

Three Dollars per Annum, in advance.

CINCINNATI:
J. T. TOLAND, PUBLISHER AND PROPRIETOR.

J. TAFT, DENTIST, NO. 56 WEST FOURTH STREET.

